# Negative Feedback Role of Astrocytes in Shaping Excitation in Brain Cell Co-cultures

**DOI:** 10.3389/fncel.2021.651509

**Published:** 2021-07-13

**Authors:** Elnaz Khezerlou, Neela Prajapati, Mark A. DeCoster

**Affiliations:** ^1^Department of Biomedical Engineering, Louisiana Tech University, Ruston, LA, United States; ^2^Institute for Micromanufacturing, Louisiana Tech University, Ruston, LA, United States

**Keywords:** astrocyte, calcium imaging, glutamate, excitotoxicity, neurons, blood brain barrier

## Abstract

Glial cells play an important role in maintaining neuronal homeostasis and may thus influence excitability in epileptogenesis. These cells in the brain have glutamate (Glu) transporters, which remove this neurotransmitter from the extracellular space. Lack of negative (−) feedback makes local neuronal circuits more excitable and potentially contributing to epileptogenic phenomena. In this study, the role of glial cells in providing (−) feedback is shown through different models of brain cells in culture imaged for intracellular calcium concentration [(Ca^2+^)_i_]. Moreover, here we study the individual cells by putting them in categories. Neuronal networks with high and low (−) feedback were established by using anti-mitotics to deplete glial cells. Separate stimuli with very low subthreshold concentrations of Glu (250–750 nM) were added to cultures to test if the order of stimulations matter in regard to calcium dynamics outcomes. Additionally, KCl and ATP were used to stimulate glial cells. We found that for cultures high in (−) feedback, order of the stimulus was not important in predicting cellular responses and because of the complexity of networks in low (−) feedback cultures the order of stimulus matters. As an additional method for analysis, comparison of high (−) feedback cultures, and pure astrocytes was also considered. Glial cells in pure astrocyte cultures tend to be larger in size than glial cells in high (−) feedback cultures. The potential effect of (−) feedback at the blood brain barrier (BBB) was also considered for the inflammatory responses of nitric oxide (NO) production and [Ca^2+^]_i_ regulation using brain microvascular endothelial cells (BMVECs). The inflammatory and calcium signaling pathways both indicate the negative feedback role of astrocytes, poised between the BBB and structures deeper within the brain, where neuronal synapses are homeostatically maintained by glial uptake of neurotransmitters.

## Introduction

Glial cells are generally involved in maintaining brain activity homeostasis and providing negative feedback in the central nervous system (CNS). Astrocytic cells have numerous protective roles in the CNS. For example, buffering of ions (Walz, 2000; D’Ambrosio et al., 2002), neurotransmitter uptake and synthesis (Danbolt, 2001; Kaczor et al., 2015), controlling cerebral blood flow (Abbott et al., 2006; Newman, 2015), and immunomodulation (Dong and Benveniste, 2001). These cells are the main regulators of many cellular processes and considered as support cells for neurons in the brain (Deemyad et al., 2018). By providing elasticity, they neutralize complexity of neuronal responses to different kinds of stimulations. Unbalanced excitation-inhibition activities in the brain are the major reason for epileptic seizures. In the absence of astrocytes or under pathological conditions which leads to hyperreactivity of these cells, lack of CNS homeostasis can occur which results in disease including epilepsy (Becerra and Cardona, 2017; Rosch and Samarut, 2019; Van Horn and Ruthazer, 2019).

Glutamate is the major excitatory neurotransmitter of the brain and this transmitter is involved in numerous neurodegenerative diseases such as stroke, epilepsy, and CNS traumatic injury (Zhao et al., 2019). Throughout these pathological events, excessive amounts of glutamate results in over-stimulating the receptors of this neurotransmitter and causes glutamate excitotoxicity which leads to increased Ca^2+^ influx and finally neuronal death (Olloquequi et al., 2018). Balancing excessive amounts of glutamate by astrocytes will help the normal functioning of the CNS. Several studies have found that pathological conditions can result in decreasing the expression of glutamate transporters, reducing the capacity of glutamine synthase, and decreasing the function of potassium channels, all of which are important functions in astrocytes (Kielbinski et al., 2016; Pekny et al., 2016; Garwood et al., 2017; Oschmann et al., 2018). Thus, dysfunction of astrocytes can lead to many neurological diseases that are associated with glutamate-induced stress to neurons.

A growing body of evidence suggests that inflammation in brain cells and peripheral immune cells contribute to both the onset of individual seizures and epileptogenic processes (Vezzani et al., 2011; Rana and Musto, 2018; Semple et al., 2020). Excessive increase in NO is a major inflammatory response in the body which occurs due to formation by inducible nitric oxide synthase (iNOS), normally absent in a resting cell. The iNOS results in sustained release of NO over time at high levels leading to formation of numerous reactive nitrogen oxide species (RNOS) responsible for a wide spectrum of physiological and pathological effects (Thomas et al., 2008). Throughout initiation and progression of epilepsy, formation of reactive oxygen species (ROS) occurs (Zhu et al., 2017; Hu et al., 2019; Terrone et al., 2020) which react with NO to form peroxynitrite (ONOO-), a toxic RNOS molecule that causes oxidation of bioactive molecules, such as proteins, lipids, and nucleic acid, and can cause neuronal death (Shin et al., 2011; Hu et al., 2019).

**SCHEME 1:**
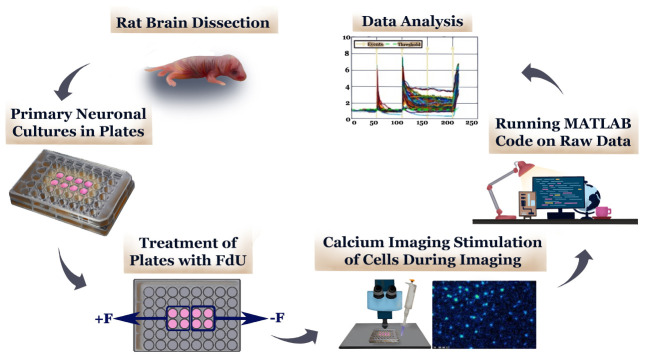
Calcium Imaging of high and low (−) feedback neuronal cell cultures. The anti-mitotic FdU (5-Fluoro-2-deoxyuridine) was used to isolate neurons in cell cultures by depleting glial cells. Half of each plate was treated with FdU (+F), and the other half treated with normal cell culture media (−F).

In this study, we investigated the role of astrocytes in providing negative feedback in predicting calcium response of primary neurons from the cortex region of the brain. We stimulated different cultures with both glutamate and KCl, because astrocytes are known to buffer both of these substances in the brain (Walz, 2000; Danbolt, 2001). We demonstrated that calcium responses for neuronal networks low in negative feedback (cultures containing 50% astrocytes by immunostaining), are more complex and less predictable compared to those high in negative feedback (cultures containing >70% astrocytes by staining). An outline in Scheme 1 displays a workflow diagram for the depletion of glial cells from neuronal cultures, real time calcium imaging, and data analysis of data sets of different cultures including high and low negative feedback cultures which were used in these studies. Moreover, in this study we investigated the role of astrocytes in modulating the NO and [Ca^2+^]_i_ response from the brain microvascular endothelial cells (BMVECs) highlighting the excessive NO synthesis and [Ca^2+^]_i_ influx in BMVECs during inflammation and considering their very close apposition at the BBB.

## Materials and Methods

### Cortical Cell Culture

For obtaining cortical cells, dissection of newborn rats with the age of 1 or 2 days was carried out. After decapitating the rats, the brain tissue was placed in dissecting solution which consisted of Basal Media Eagle (BME, Sigma) with 0.5% Penicillin/Streptomycin (PS) (Sigma). Then, the meninges were removed under the microscope. All procedures were performed adhering to the protocol approved by the Louisiana Tech University Animal Care and Use Committee. After completing dissection, the cortical tissue was triturated three times using trypsin and Neuronal Culture Media (NCM) containing BME, Ham’s F-12 K media without L-Glutamine (ATCC), 10% Horse Serum (Sigma-Aldrich), 10% Fetal Bovine Serum (Sigma-Aldrich), and supplemented with glucose, glutamine and PS as previously described (Daniel and DeCoster, 2004). The cells were then mechanically isolated by trituration and allowed about 10 min to form a neuronal cell supernatant. This supernatant was stored in a 15 mL tube on ice after each trituration. The neuronal cell supernatant was then centrifuged to form a pellet. After obtaining the cell count, cells were then plated in a 48 well culture plate (cell culture treated, Cellstar) at a density of 1 million cells per well and maintained in a 5% CO_2_ and 37°C humidified incubator. After 5 days *in vitro*, one half (+ F side) of the cell culture plate was treated with 4 μM of the anti-mitotic 5-Fluoro-2-deoxyuridine (FdU) (Sigma-Aldrich) to isolate neurons in cell cultures by depleting glial cells. The remaining half (−F side) was supplemented with warmed NCM (Hui et al., 2016; Kelly et al., 2016).

As a new method for culturing mixed brain cell cultures, the NCM for −F side in cortical plates was removed and replaced with Astrocyte Culture Medium, promoting glial cell proliferation. Exchanges of culture medium were performed on the cultures every 2–3 days.

### Primary Astrocytes Culture

Primary astrocytes were obtained after dissection of rat brain cortex (Wang et al., 2005). These cells were grown in flasks and were not treated with FdU. For growing astrocytes, astrocyte culture medium containing Ham’s F12 K media with L-Glutamine, 5% Horse Serum, and 5% Fetal Bovine Serum was used. Upon reaching the high density of growth, cells were lifted from flasks and dissociated with trypsin/EDTA (Sigma), centrifuged to a pellet, and were then plated into a 48 well cell culture plate coated with poly-lysine (PLL, Sigma) at an optimal density of 3,000 cells per well (Daniel and DeCoster, 2004). Primary cultures of astrocytes contained at least 95% astrocytes as determined by glial fibrillary acidic protein (GFAP) staining (Gao et al., 2013), with a minority (5%) content of microglia (Wang et al., 2005).

### Brain Microvascular Endothelial Cell (BMVECs) Culture

Brain microvascular endothelial cells were isolated from the primary glial culture obtained after dissection of rat brain cortex. The glial cultures were treated with 5.5 μM of puromycin dihydrochloride to kill all other cell types in the culture except for the endothelial cells. Endothelial cells are encoded with a puromycin N-acetyl transferase gene (PAC gene), which confers resistance to the action of puromycin (Saleh et al., 2020). Thus, obtained cells were then grown in Rat Endothelial Growth Media (Cell Applications, Inc) with 6% Rat Endothelial Growth Factor and incubated at 5% CO_2_ and 37°C. The BMVECs were characterized by staining them against Von Willebrand Factor (VWF, Supplementary Figure 1A), a specific marker of the endothelial cells (Zanetta et al., 2000). BMVECs exhibit a higher proliferation rate than the astrocytes (Bernard-Patrzynski et al., 2013). Therefore, 10,000 (10k) cells per mL cell density of BMVECs and 20,000 (20k) per mL of astrocytes were plated in 48 well cell culture plates (Griener) to maintain the confluency for both cell types at approximately same level over the time of study. However, we did observe with time after stimulus that BMVECs continued to outpace the astrocytes as an increasing percentage of the cell population.

### Co-Culture of Astrocytes and BMVECs

Astrocyte and endothelial cells were plated at a cell density of 1:1 in the 48 well cell culture plates with in the 1:1 mixture of astrocyte growth media and rat endothelial growth media. Cells were plated at the total density of 20k cells per mL. The cells were characterized by staining the astrocyte cells against glial fibrillary acidic protein (GFAP) (Olude et al., 2015) amongst the nuclei of endothelial cells which were stained by 4,6-diamidino-2-phenylindole (DAPI) (Supplementary Figures 1B,C). The quality of co-culture model was determined by analyzing the GFAP stained co-culture images using Image Pro 7 software. BMVECS covered twice as much area as the astrocyte cells in the co-culture model, which still allowed for the influence of astrocytes on overall cell responses to be observed.

### [Ca^2+^]_i_ Imaging

Imaging of cortical neuronal cultures was performed after 10–12 days *in vitro* (DIV). DIV for each cell type was selected based on growth rate. The cells were incubated in a loading solution of the calcium indicator Fluo-4 AM (Invitrogen) for 1 h. Then, using warmed Locke’s solution, the cells were washed and recovered and re-incubated for 30 min. While the cells were recovering, the materials (different concentrations of fresh Glutamic Acid (Glu), KCl, ATP, and Ionomycin) were prepared in Locke’s solution. For imaging the cells, an Olympus CKX41 inverted microscope with a 488 excitation wavelength filter in a frame rate of 4 s per frame with Intracellular Imaging software (InCyt Im Imaging system, version 5.29e, Cincinnati, OH) was utilized. A baseline (recording of cells without treatment) of 50 frames was recorded, and stimulations were added to the experiment at prearranged intervals (50, 100, and 150 frames). These 3 designated time points corresponded to events 1, 2, and 3, respectively. Stimulations were added without washing out the media between additions. Ionomycin was used as a positive control (event 4) for the experiments, which was added at frame 200. The same protocol was used for imaging of other cell types (DeCoster et al., 2002; Kelly et al., 2016).

For the BMVECs, astrocytes and the co-culture model pre-treated with inflammatory stimulus, the cells were stimulated using ATP (100 μM) and glutamate as indicated after the baseline recording of 15 frames. Peak [Ca^2+^]_i_ response was analyzed for the peaks obtained for ATP stimulation, as no significant response was found in most cells for glutamate stimulation at the same concentration. The [Ca^2+^]_i_ peaks were calculated by analyzing data obtained after ROI selection and fluorescent intensity measurement per ROI.

### ROI Selection and Measurement of Fluorescence Intensity

Intracellular imaging software (InCyt Im Imaging system, version 5.29e, Cincinnati, OH) was used to generate regions of interest (ROIs) around every cell in the data set after the experiment. ROIs were created around cells on the basis of responses to stimulations and using Ionomycin as a positive control stimulus. Using these ROIs, fluorescence intensity over time per ROIs was measured. In addition to InCyt Im software, we also used MATLAB codes which were written specifically for us to categorize the cells. At least five different platings from at least two different primary culture preparations were used for every condition reported in this work. Later, these ROIs and pictures from calcium imaging were used to do image analysis. In this study, only cells showing at least a 1.2-fold (20%) increase in fluorescence compared to baseline (time 0 or T_0_) values (which were normalized to 1), were considered as Responders. Cells showing less than 20% increase for any of the stimulations were considered as Non-Responders. We found that astrocytes did not show [Ca^2+^]_i_ responses to lower concentrations of glutamate (250–500 nM). Astrocytes in the cultures were identified on the basis of [Ca^2+^]_i_ responses to KCl and ATP stimulations. In separate experiments, DAPI staining and Diffquik staining was performed to understand the networks of ROIs in cultures (Kelly et al., 2016). Additionally, DAPI was used for cell counting and for calculating nuclear area and the nuclear area factor (NAF) (DeCoster, 2007), comparing cultures with high and low (−) feedback.

### Inflammatory Stimulus Treatments

Brain microvascular endothelial cells, astrocytes and co-culture of these two cells were plated in 48 well cell culture plates at the density of 10, 20, and 20k cells/ml, respectively and incubated at 5% CO_2_ and 37°C. The cells were treated with an inflammatory stimulus, a combination of 100 ng/ml of Tumor Necrosis Factor (TNF) (Sigma Aldrich) and 5 μg/ml of Lipopolysaccharides (LPS) (Sigma Aldrich) when they reached ∼30% confluency. After treatment, cells were incubated up to 6 days *in vitro* (DIV) to assess the NO concentration in the wells on 2, 4, and 6 DIV. The experiment was reproduced for studying [Ca^2+^]_i_ responses in each culture using agonists ATP (100 μM), glutamate (100 μM), and Ionomycin (1 μM) stimulation. The cells were stimulated and imaged in real time using the described calcium imaging system 4 DIV after treatment with inflammatory stimulus.

### Nitric Oxide Assay

For quantitative colorimetric determination of Nitric Oxide, Invitrogen Griess Reagent Kit (#LSG-7921) for nitrite quantification was obtained from Thermo Fisher Scientific and used according to standard protocols. A total of 100 μl of media each, from the wells after treatment was reacted with Griess reagent to measure the stable nitrates or nitrites for quantification of NO generated in the sample (Wang et al., 2012). The absorbance reading of these stable byproducts of NO was obtained by using a Beckman Coulter DU 800 spectrophotometer. A standard curve of 0, 5, 25, and 50 μM of sodium nitrite obtained from the reagent kit was prepared to obtain the absorbance of the standard nitrite solution. The standard curve equation (which ranged from 0.989 < R < 1) was used for each experiment to calculate the values of NO concentrations in Microsoft Excel using the absorbance readings.

### Immunohistochemistry Assay

Staining of the high and low (−) feedback cultures for Glial Fibrillary Acidic Protein (GFAP) was carried out after calcium imaging of these cultures. After calcium imaging experiment, the whole plate was fixed using ice-cold methanol and 1× PBS and stored in 2–8°C (in refrigerator). Later the PBS was removed from pre-fixed cells and 0.2% Triton X 100 (in 1× PBS) was added to cover the whole surface and kept at room temperature for 15 min. Then, Triton X 100 was removed, and 2% Goat Serum (in 1× PBS) was added to cover the surface. The plate was sealed with parafilm and kept at 2–8°C (in refrigerator) for 4–5 h or overnight. After keeping the plate for 4–5 h or overnight, goat serum was removed and Primary Antibody (1° Ab) (Anti-GFAP produced in rabbit, Sigma-Aldrich, in 1:500 1× PBS) was added to cover the whole surface. The plate was sealed with parafilm and kept at 2–8°C (in refrigerator) for 24 h or overnight. After 24 h, 1° Ab was removed and washed twice with 1× PBS. Secondary Antibody (2° Ab, Thermo Fisher Scientific, 1:500 Goat Anti- Rabbit Ig Ab in 2% Goat Serum which was in 1× PBS), was added to cover the whole surface. The plate was sealed with parafilm and covered with aluminum foil and kept at room temperature for 45–60 min. Then, 2° Ab was removed and washed twice with 0.2% Triton X 100 (in 1× PBS). Lastly 1× PBS was added to cover the surface. After this final step, fluorescence microscopy was used to observe and capture staining using digital microscopy (Leica).

## Results and Discussion

### Orders of Different Stimulations (Glu-First or KCl-First) Is Important in Predicting Cellular Responses

**FIGURE 1:**
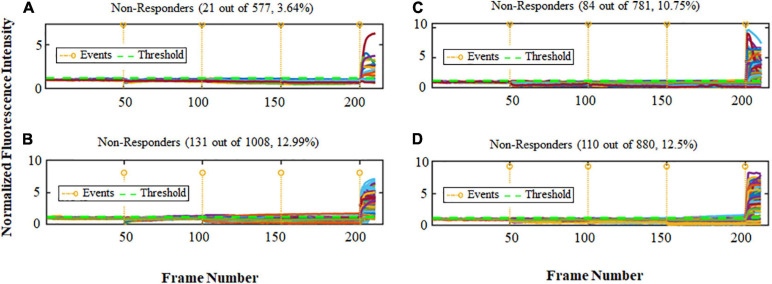
Ca^2+^ peak intensity obtained for cultures with high and low (–) feedback. The horizontal axis is the frame number indicating time periods with total range of 4 sec each (1 frame no. = 4 s). Total time for each experiment was thus approximately 14 min. The vertical axis shows the normalized values for fluorescence intensity corresponding to calcium activity. The orange lines show events (total of 4) and the green line shows threshold (1.2). Note that non-responder cells did not show a response to any of the first 3 stimuli, however they all did respond to Ionomycin (Iono, positive control, 4th event). **(A)** Non-responder cells for Glu-first stimulus of low (–) feedback cultures. Events = Glu 250 nM, KCl 50 mM, Glu 500 nM, and Iono. **(B)** Non-responder cells for Glu-first stimulus of high (–) feedback cultures. Events = Glu 250 nM, KCl 50 mM, Glu 500 nM, and Iono. **(C)** Non-responder cells for KCl-first stimulus of low (–) feedback cultures. Events = KCl 50 mM, Glu 250 nM, KCl 50 mM, and Iono. **(D)** Non-responder cells for KCl-first stimulus of high (–) feedback cultures. Events = KCl 50 mM, Glu 250 nM, KCl 50 mM, and Iono.

To define the importance of order of stimulus inputs in predicting the cellular response for both cultures with high and low (−) feedback, calcium imaging of cortical cells was carried out and analysis of calcium response for a large dataset of 3,246 cells was performed. Two different sets of experiments have been conducted for each high and low (−) feedback cultures, one set starting with Glu (250 nM) and KCl (50 mM) followed by a final Glu (500 nM) stimulus, and the other set starting with KCl (50 mM) and Glu (250 nM) followed by a final KCl (50 mM) stimulus. The percent of non-responder cells in each condition can help us determine the activity of the network. We found major differences in the two types of cultures. The percent of non-responders for low (−) feedback cultures in Glu-first experiments was 3.57 fold lower than high (−) feedback cultures (a large 257% comparative difference) as is shown in Figures 1A,B. In contrast, the percent of non-responders for KCl-first experiments (expected to also stimulate glia), was much more similar (only a 16% comparative difference) for both high and low (−) feedback cultures (Figures 1C,D) meaning that cells are more equally responsive to these stimulations than for Glu in high (−) feedback culture. This demonstrates the effect of the glial cells in balancing the activity in brain cell networks in high (−) feedback cultures, where more astrocytes are present.

Next, we compared the cells in each culture which responded to all of the events in Glu-first (250 nM) and KCl-first (50 mM) conditions. We found that for low (−) feedback cultures the percentage of cells that responded to all stimulations of Glu-first condition are more than for high (−) feedback cultures. In addition, the percentage of cells that responded to all three stimulations of KCl-first condition are more than low (−) feedback cultures. This is because of the differences in amount of the neurons and glial cells which are present in these cultures and consequently the importance of the glial cells in providing negative feedback to balance the system. Table 1 shows the data for these comparative experimental conditions of percentage of cells which responded to all events.

**TABLE 1:** Percentage of cells that responded to All of the stimulatory events.

	Glu-first (%)	KCl-first (%)
Low (−) feedback	38.82	5.50
High (−) feedback	20.44	12.95

*Glu-first events = Glu 250 nM, KCl 50 mM, Glu 500 nM, and Iono. KCl-first events = KCl 50 mM, Glu 250 nM, KCl 50 mM, and Iono. Glu-first low (−) feedback: *n* = 577 cells; Glu-first high (−) feedback: *n* = 1008 cells; KCl-first low (−) feedback: *n* = 781 cells; KCl-first high (−) feedback: *n* = 880 cells. Chi-Square analysis result shows *p* < 0.001.*

After analyzing these large data sets of thousands of cells, we found that the percentage of cells which responded to only first and second events, only second and third events, only first and third events and all of the events for Glu-first order was higher for low (−) feedback cultures which include more neuronal cells. It demonstrates that the neurons are more responsive to Glu. On the other hand, this percentage for KCl-first order shifted in comparison to Glu-first experiments, and was higher for high (−) feedback cultures than low (−) feedback cultures. The data are shown in Table 2.

**TABLE 2:** Percentage of cells that responded to only events 1 and 2, only events 2 and 3, only events 1 and 3, and all of the stimulatory events.

	Glu-first (%)	KCl-first (%)
Low (−) feedback	81.97	37.38
High (−) feedback	55.75	50.45

*Glu-first events = Glu 250 nM, KCl 50 mM, Glu 500 nM, and Iono. KCl-first events = KCl 50 mM, Glu 250 nM, KCl 50 mM, and Iono. Glu-first low (−) feedback: *n* = 577 cells; Glu-first high (−) feedback: *n* = 1008 cells; KCl-first low (−) feedback: *n* = 781 cells; KCl-first high (−) feedback: *n* = 880 cells. Chi-Square analysis result showed *p* < 0.05.*

**FIGURE 2:**
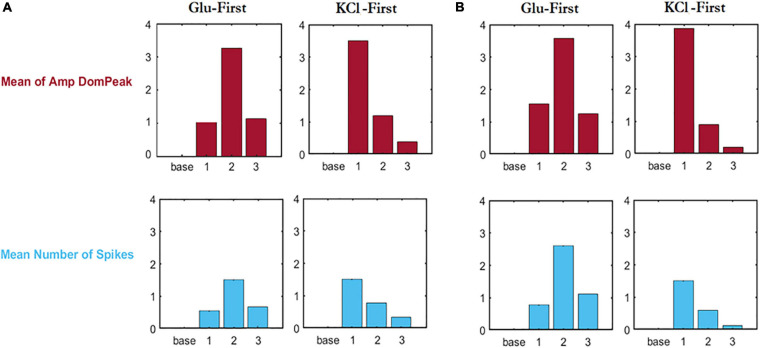
Comparison of Mean number of spikes (blue) and Mean of amplitude of dominant peak (red) for different conditions of total 3,246 cells. The horizontal axis shows different events (base = baseline, followed by stimuli 1–3) which for Glu-first are: Glu 250 nM, KCl 50 mM, and Glu 500 nM. The events for KCl-first are: KCl 50 mM, Glu 250 nM, KCl 50 mM. **(A)** Analysis for cultures high in (–) feedback. **(B)** Analysis for cultures low in (–) feedback.

Moreover, comparison of Mean number of spikes and Mean of amplitude of dominant peak was carried out for each of the different combination of orders of 3,246 cells in both high and low (−) feedback cultures (Figure 2). In contrast to the type of responses to stimulus events, data in Figure 2 show that the amplitude of responses, and number of spike events, were quite similar for all combinations. However, this analysis did reveal patterns of amplitude responses for the different sequences of stimulus. Thus, for cultures high in (−) feedback (Figure 2A), the mean amplitude of dominant peak for KCl-first (50 mM) conditions was different than Glu-first (250 nM) conditions in a way that after first KCl stimulus, cells were desensitized, and we observed less amplitude for second stimulus which was Glu (500 nM) and for third stimulus the amplitude was almost 1/4 of Glu. This desensitized response was even less for low (−) feedback cultures (Figure 2B) after KCl stimulation first, since fewer glial cells were present. Quantification of the mean number of spikes also showed a higher number of spikes for low (−) feedback cultures (Figure 2B) compared to high (−) feedback cultures (Figure 2A) for Glu-first stimulation experiments.

Another set of experiments was conducted with a novel culturing method where we replaced the NCM with astrocyte media to model proliferation of pure astrocytes in mixed neuronal cultures. One set started with Glu (500 nM) and KCl (50 mM) followed by a final Glu (750 nM) stimulus, and the other set started with KCl (50 mM) and Glu (500 nM) followed by a final KCl (50 mM) stimulus. For these cultures a total of 3,626 cells were analyzed. We found that the non-responder cells for high (−) feedback cultures was about 5.96 fold higher than for low (−) feedback cultures for Glu-first orders and 4.48 fold higher than for KCl-first orders. This was interpreted to indicate that additional astrocyte media helped proliferation of more glial cells in high (−) feedback cultures and consequently the ratio of non-responder cells was higher for these cultures. Data for the non-responder category of these experiments is shown in Table 3.

**TABLE 3:** Percentage of non-responder cells to different orders of stimulation.

	Non-responder cells (%)
Glu-first, low (−) feedback	2.93
Glu-first, high (−) feedback	17.48
KCl-first, low (−) feedback	4.09
KCl-first, high (−) feedback	18.33

*Glu-first events = Glu 500 nM, KCl 50 mM, Glu 750 nM, and Iono. KCl-first events = KCl 50 mM, Glu 500 nM, KCl 50 mM, and Iono. Glu-first low (−) feedback: *n* = 237 cells; Glu-first high (−) feedback: *n* = 1247 cells; KCl-first low (−) feedback: *n* = 660 cells; KCl-first high (−) feedback: *n* = 1446 cells. Chi-Square analysis result showed *p* < 0.01.*

### Repeated Glu at the Same Concentration Gives Different Responses Which Shows Order of Glu Concentrations Matter in Different Types of Networks

**FIGURE 3:**
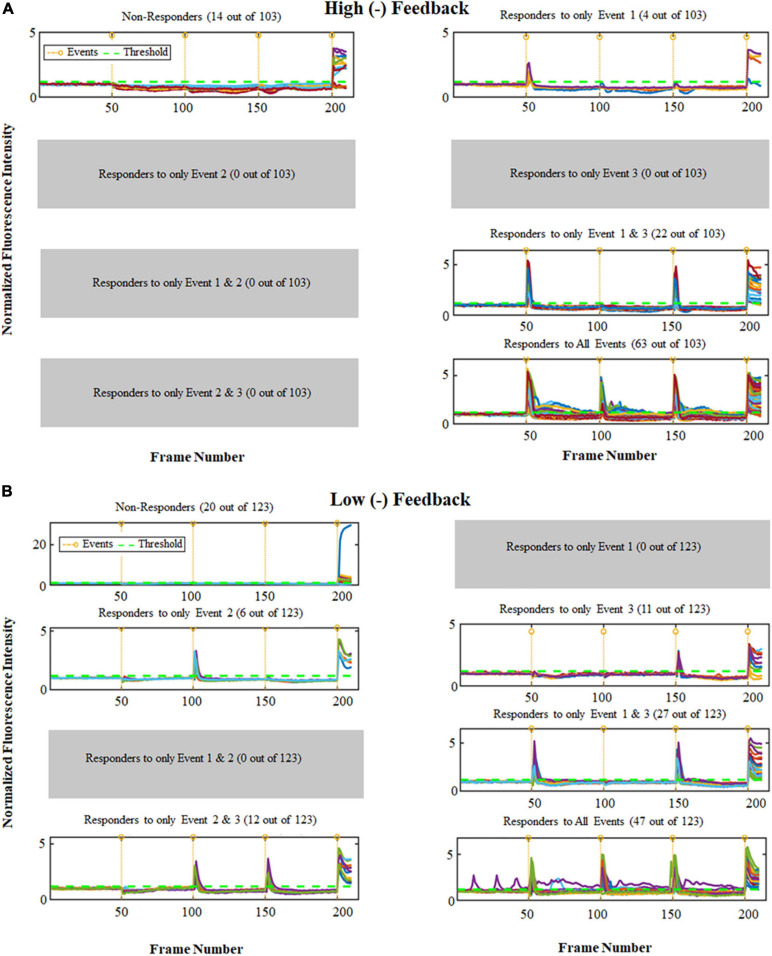
Ca^2+^ peak intensity obtained for 3 repeated Glu stimuli (500 nM) for cultures with high and low (–) feedback. The horizontal axis is the frame number indicating time periods with total range of 4 s each (1 frame no. = 4 s); total recording time for each experiment was thus about 14 min. The vertical axis shows the normalized values for fluorescence intensity corresponding to calcium activity. The vertical orange lines show events, and the green horizontal line shows Threshold (1.2). Note that Non-Responder cells did not show a response to any of the first 3 stimuli, however they all did respond to Ionomycin (positive control, added at 4th stimulus). **(A)** Analysis for cultures high in (–) feedback. **(B)** Analysis for cultures low in (–) feedback. Chi-Square analysis result shows *p* < 0.0001.

In experiments using the same Glu stimulus (500 nM) up to 3 times in the same network of cortical cell cultures, those with high (−) feedback remained highly predictable. For these experiments a total of 226 cells were analyzed. For cultures high in (−) feedback, 61% of cells (*n* = 63 responders out of 103 cells) showed a response to all three stimuli. Whereas, for cultures low in (−) feedback, 38% of cells (*n* = 47 responders out of 123) showed a response to all stimuli. A thorough analysis of these experiments is shown in Figure 3, and the calcium response for both types of cultures are different in terms of number of the cells in different combination of events. Cultures high in (−) feedback have no responder cells in four of the possible 8 stimulus conditions, those being: for only event 2, only event 3, only event 1 and 2, and only event 2 and 3. This represents 50% of the possible outcomes with no responses and demonstrates the simplicity of network conditions in these cultures. On the other hand, cultures low in (−) feedback have more cells which responded in different combination of events (6 of the 8 possible outcome categories), which shows the complexity of networks formed and less predictability of these networks. In this case, only 25% of the possible outcomes remained unoccupied (Figure 3). These results validate the hypothesis that the order of concentrations of Glu matters as we have a shift in the population responses for the different culture environments (high and low negative feedback).

### Detecting Cells (Glial Cells and Neurons) in Calcium Analysis Networks

To detect the glial cells from neurons in mixed cultures with high and low (−) feedback, different orders of Glu and KCl have been tested to determine if there were any difference in calcium response. A total of 3,246 cells were analyzed for these comparative experimental conditions. Two different sets of experiments have been conducted for high and low (−) feedback cultures, one set starting with Glu (250 nM) and KCl (50 mM) followed by a final Glu stimulus (500 nM), and the other set starting with KCl (50 mM) and Glu (250 nM) followed by a final KCl (50 mM) stimulus. Previous studies have shown that the expression of calcium channels in astrocytes depends on the presence of neurons (Corvalan et al., 1990). Based on our observations, there was a difference in the percent of responders that only responded to event 2 of Glu-first order and the responders that only responded to event 3 of KCl-first order. Supplementary Table 4 shows that for Glu-first order, the percentage of cells that responded to only event 2 is 2.26 fold higher for high (−) feedback cultures. Based on previous studies the [Ca^2+^]_i_ increase observed during stimulation with K^+^ in astrocytes was because of the release of Ca^2+^ from [Ca^2+^]_i_ stores after activation of G-protein-linked receptors caused by neurotransmitter (Glu) release from depolarized synaptic terminals (Carmignoto et al., 1998). It demonstrates that the cells which showed [Ca^2+^]_i_ increase on only event 2 for Glu-first (250 nM) order and on only event 3 for KCl-first (50 mM) order may be the astrocytes which have been activated after Glu stimulation. This percentage for KCl-first order was 1.6 fold higher for high (−) feedback cultures and was 2.25-fold higher for high (−) feedback cultures in the Glu-first experiments.

In another set of experiments, astrocyte proliferation was promoted, by repeatedly replacing NCM with Astrocyte media as described in methods section. We found that the percentage of cells which responded to only event 3 for KCl-first order was similar to the results in Supplementary Table 4, with only a 1.03% response for high (−) feedback cultures. This percentage was even lower for low (−) feedback cultures, at 0.3%.

**FIGURE 4:**
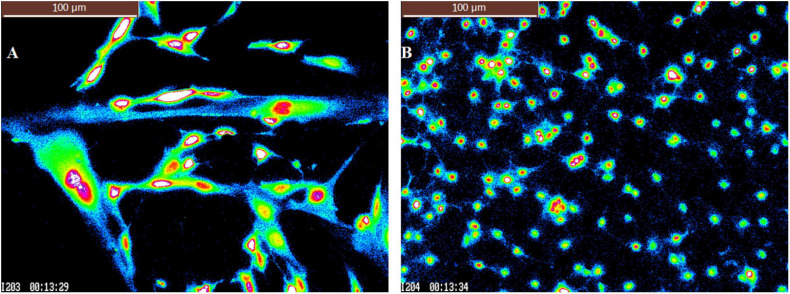
Pseudocolor pictures of calcium imaging for **(A)** pure astrocytes. **(B)** High (–) feedback culture. Scale bar = 100 microns in both figure panels. “I” in bottom left of each panel = image # from the recording and elapsed time of the recording is shown in minutes and seconds (13:29 = 13 min and 29 s, for example).

As an additional method to distinguish glial cells and neurons in these studies, the [Ca^2+^]_i_ based region of interest (ROI) cell size and total cell count for neuronal cultures and astrocytes have been compared for cells used in these studies. For neuronal culture [High (−) feedback], the average [Ca^2+^]_i_ based ROI size for *n* = 153 cells was 192 ± 75 pixels. On the other hand, for astrocyte cultures, the average ROI size for *n* = 50 cells was 1,907 ± 665 pixels. The average Ca^2+^ based ROI size of astrocyte cultures was thus 9.9 times that of neuronal culture. It demonstrates that the glial cells in pure astrocyte cultures are much bigger in size (9.9-fold larger) than the responsive cells in neuronal cultures as is shown in Figure 4.

**FIGURE 5:**
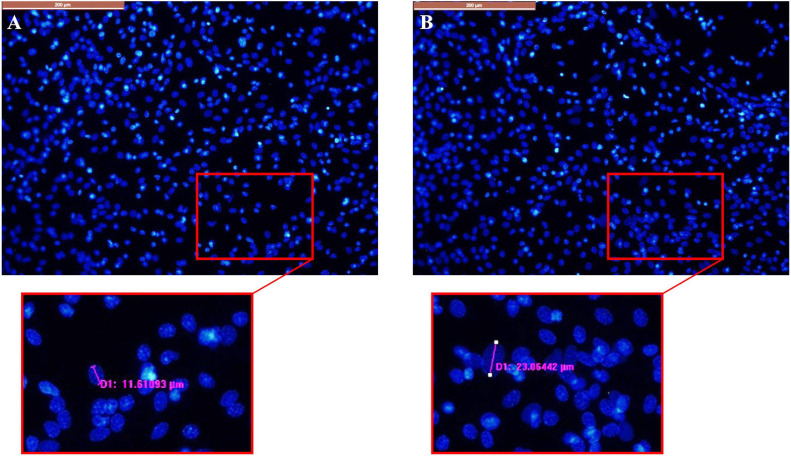
DAPI pictures of **(A)** low (–) feedback culture. **(B)** High (–) feedback culture. Scale bar = 200 μm. D1 = example diameter.

Later, we did Diffquik and DAPI staining on these cultures. Based on image analysis results of Diffquik pictures of high and low (−) feedback cultures, there was about a two-fold difference for average cell area between the two types of cultures. Average cell area for Diffquik based analysis of high (−) feedback cultures was 801 ± 55 pixel square, while for low (−) feedback cultures it was 452 ± 11 pixel square. Moreover, we analyzed the DAPI pictures of these cultures for determining the nuclei size (length). Average nuclei size for *n* = 20 cells of high (−) feedback cultures was 20.1 ± 3.93 μm and for *n* = 20 cells of low (−) feedback cultures was 11.89 ± 1.29 μm. It demonstrates that in high (−) feedback cultures the nuclei size is bigger (by about 70%) than low (−) feedback side (Figure 5).

These data were also supported by measurements of nuclear area, cell number and the nuclear area factor (NAF) comparing high and low (−) feedback cultures (Supplementary Figure 2).

**FIGURE 6:**
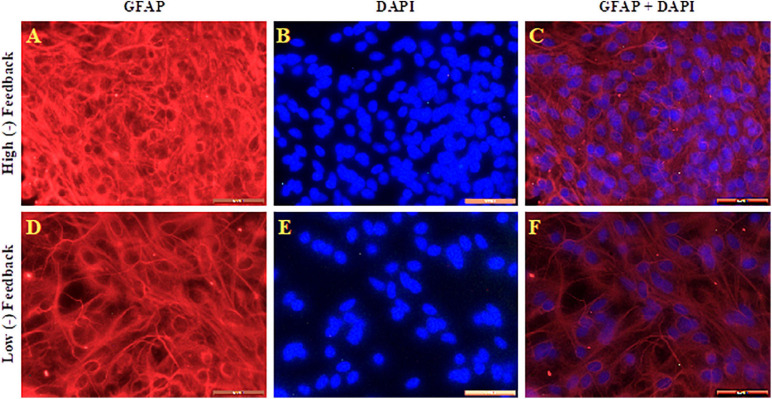
GFAP (red: **A,D**), DAPI (blue: **B,E**), and merged pictures **(C,F)** of high and low (–) feedback neuronal/astrocyte cultures, as indicated. Scale bar = 40 μm.

We carried out GFAP immunohistochemistry to determine the differences between high and low (−) feedback cultures. Figure 6 shows GFAP, DAPI, and merged pictures of GFAP and DAPI in high and low (−) feedback cultures. It is apparent in the images that, high negative feedback cultures have more glial cells and more nuclei per field of analysis than low negative feedback cultures. In contrast, low negative feedback cultures have less glial cells and nuclei per field of analysis, and more fine astrocytic processes (indicated by GFAP staining, Figure 6D).

**FIGURE 7:**
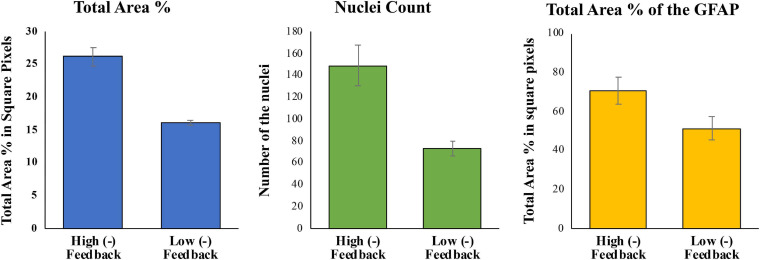
Analysis of total area and nuclei count of DAPI, and total area percent of GFAP for high and low (–) feedback cultures as indicated. Data represent *n* = 7–12 for each condition, with standard deviation error bars indicated.

Figure 7 shows quantified differences between total area and nuclei count for DAPI and total area percentage for GFAP staining of high and low (−) feedback cultures. The analysis shows that in high negative feedback cultures, a cell network was established with a greater percentage of astrocytes and greater area of GFAP positive astrocytic processes (>70%) compared to low negative feedback cultures (50% GFAP area).

### Sensitivity of Astrocytes to Glutamate

**FIGURE 8:**
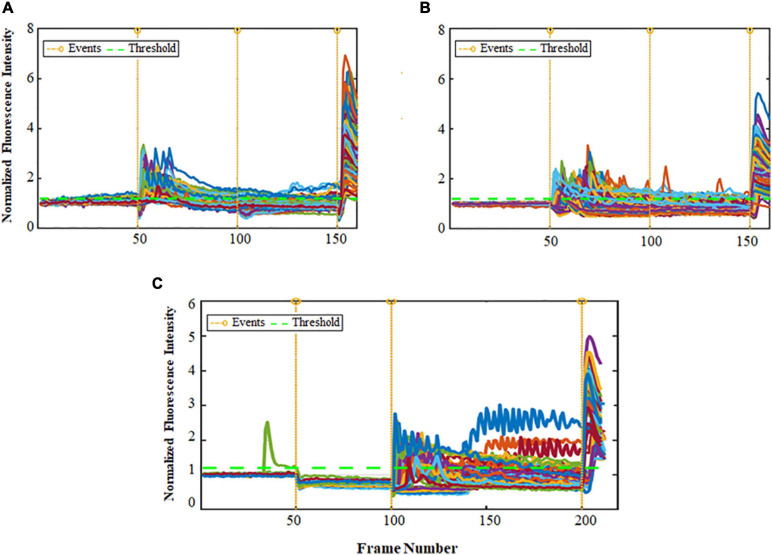
Ca^2+^ peak intensity obtained for 3 stimulations of ATP, Glu and Ionomycin (Iono) for pure astrocyte cultures. The horizontal axis is the frame number indicating time periods with total range of 4 s each (1 frame no. = 4 s); and the vertical axis shows the normalized values for fluorescence intensity corresponding to calcium activity. The orange vertical lines show events (3) and the green horizontal line shows threshold (1.2). **(A)** Events: ATP 100 μM, Glu 100 μM, and Iono. **(B)** Events: ATP 100 μM, Glu 5 mM, and Iono. **(C)** Events: Glu 5 mM, ATP 100 μM, and Ionomycin.

Glial cells are less sensitive to Glu in comparison to neuronal cells, and in fact, protect neurons from glutamate excitotoxicity (Cornell-Bell et al., 1990; Jensen and Chiu, 1991; Rosenberg et al., 1992; Becerra and Cardona, 2017). For testing sensitivity of pure astrocyte cultures, different combinations of Glu concentrations were used as stimuli for [Ca^2+^]_i_ response. A total of 880 pure astrocyte cells were analyzed for these experiments. We had three different combinations of stimulations: (1) ATP 100 μM and Glu 100 μM, (2) ATP 100 μM and Glu 5 mM, and (3) Glu 5 mM and ATP 100 μM. For experiments that we tried ATP 100 μM as a first stimulus, we had total responder cells of 347 (out of 651). On the other hand, for experiments where Glu 5 mM was the first stimulus, only 50 cells (out of 229) responded, which was much less than ATP first response. The results demonstrate that the pure astrocytes are not sensitive to high concentrations of Glu as much as less concentrations of ATP as is shown in Figure 8. However, after comparing number of the spikes and amplitude of dominant peak for [Ca^2+^]_i_ response of Glu 5 mM as first stimulation and Glu 100 μM as first stimulation, we detected that a small percentage of these cells are sensitive to higher concentrations of Glu. The mean number of the spikes for Glu 5 mM was 2.64 fold of Glu 100 μM, and the mean amplitude of dominant peak for Glu 5 mM was 1.63 fold of Glu 100 μM.

### Role of Astrocytes in Balancing Signaling Response at the BBB

**FIGURE 9:**
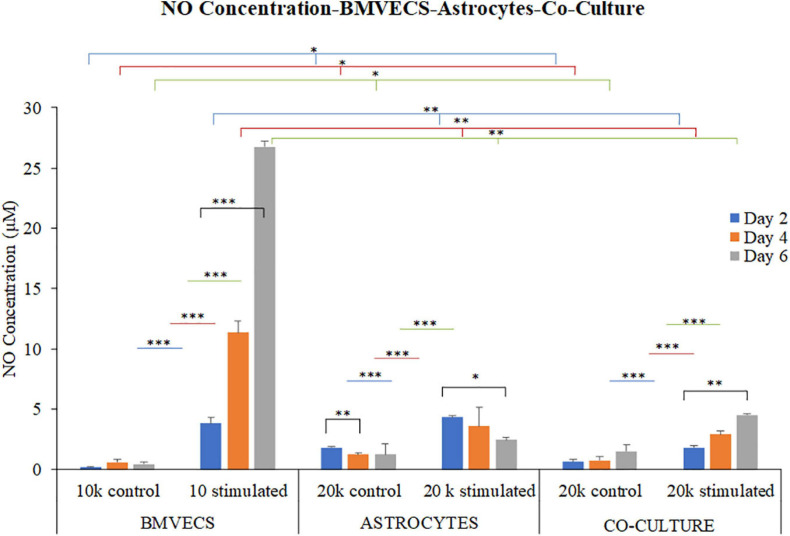
Nitric Oxide Concentration over time (2,4 and 6 DIV) after treatment with inflammatory stimulus- a combination of TNF (100 ng/ml) and LPS (5 μg/ml) for BMVECs, Astrocytes and their Co-culture Model – indicating that the presence of astrocytes provides negative feedback against the excessive NO production from BMVECs due to inflammatory stimulus in the co-culture model. The data presents an average of 3 experiments (*N* = 3) with triplicated samples (*n* = 3) per condition used for the experiment. The error bars represent SEM values and “^∗∗∗^” represents *p* < 0.001, “^∗∗^” represents *p* < 0.01, and “^∗^” represents *p* < 0.05.

We next tested for potential (−) feedback effects of astrocytes in cultures with brain microvascular endothelial cells (BMVECS) with NO and [Ca^2+^]_i_ as cellular outputs for analysis. A non-linear increase in NO synthesis was seen in BMVECs treated with inflammatory stimulus compared to the control cells with 3.5 ± 0.3 μM mean increase in NO concentration on day 2 that increased significantly to 10.5 ± 0.6 μM and 26 ± 0.3 μM by day 4 and day 6, respectively, after treatment (Figure 9). An opposite trend of NO synthesis was seen in astrocytes with NO concentration decreasing from day 2 to day 6 after treatment. Astrocytes treated with inflammatory stimulus showed an average increase in NO at 2.5 ± 0.1 μM on day 2 compared to the control cells which went down slightly to 2.3 ± 0.9 μM and 1.2 ± 0.5 μM by day 4 and day 6, respectively (Figure 9). The co-culture model shows a trend similar to BMVECs culture with NO concentration in stimulated cells going up from day 2 to day 6 after treatment, but at a markedly reduced level. Mean NO increase was seen at 1.2 ± 0.2 μM on day 2, 2.2 ± 0.3 μM on day 4 and 3 ± 0.3 μM on day 6 for the stimulated cells compared to the controls.

**FIGURE 10:**
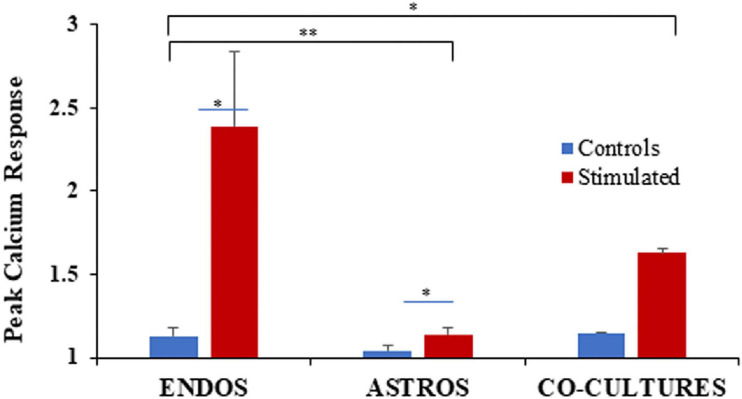
Peak calcium response analysis in control cells (blue) and cells with the inflammatory stimulus (red), for BMVECs (ENDOS), astrocytes (ASTROS), and their co-culture model. Peak analysis was carried out in response to 100 μM ATP stimulation. Peak calcium response is shown in comparison to the time 0 (T_0_) values for each individual cell as was done for all neuronal culture experiments. Data represent the averages of *n* = 3 experiments with two wells per condition, and number of samples (cells) ranging from 40 to 211. Error bars represent SEM values. * represents *p* < 0.05 and ** represents *p* < 0.01.

The presence of about 33% astrocytes in the mixed cultures, showed significantly reduced levels of NO compared to the BMVEC monoculture alone but keeping a similar trend of increased NO levels over time (2–6 days). These results thus showed that astrocytes provide negative feedback to the excessive NO synthesis shown by BMVEC cells alone. We next tested for the potential for astrocytes to balance ATP-stimulated [Ca^2+^]_i_ changes in BMVEC cultures. The analysis of [Ca^2+^]_i_ peak response for 100 μM ATP stimulation of BMVECs, astrocytes alone, and BMVEC-astrocyte co-cultures is shown in Figure 10.

For BMVECs alone, a 139% increase in the peak calcium response for cells pre-treated with inflammatory agents was measured compared to only a 13% increase for control cells. In the case of astrocytes, the peak [Ca^2+^]_i_ response for cells pre-treated with the inflammatory agents was found to be at 14% above the baseline levels, while the control cells showed only a 4% increase. In the co-culture model, the cultures pre-treated with inflammatory agents showed 63% increase in peak [Ca^2+^]_i_ response while the control cells showed 15% increase above the baseline.

These results show that [Ca^2+^]_i_ response for co-cultures of BMVECs and astrocytes pre-treated with inflammatory agents lies in between the values shown by monocultures of BMVECs and astrocytes alone. For astrocytes only, cells treated with inflammatory agents showed a peak [Ca^2+^]_i_ influx that was ∼10-fold less compared to BMVECs alone, while the astrocyte control cell response was reduced by threefold compared to BMVEC controls. The pre-treated co-cultures showed 76% inhibition compared to the BMVEC monocultures, and a 49% enhancement compared to astrocyte monoculture. Thus, the results indicated that the presence of astrocytes in co-culture models regulate the significantly high [Ca^2+^]_i_ influx shown by BMVECs during inflammatory condition.

**FIGURE 11:**
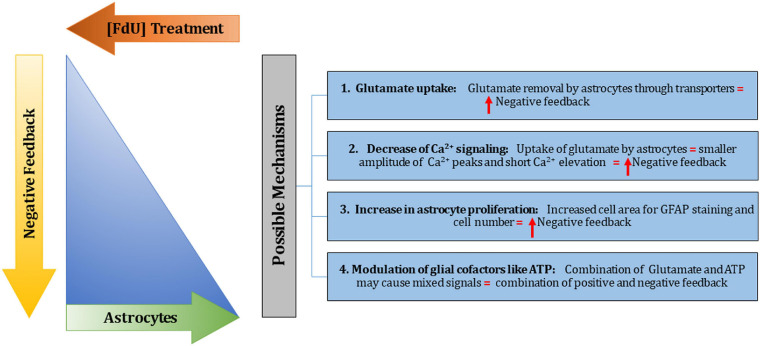
Potential mechanisms of action of negative feedback. In this *in vitro* study, astrocytes, represented by the blue triangle, were controlled by the use of the anti-mitotic FdU (indicated by FdU treatment arrow). This treatment decreases the% of astrocytes in the cultures, as indicated by the top, smaller apex of the blue triangle. If we allow astrocytes to proliferate (by not treating with FdU), then the% of astrocytes increases in the cell culture, as indicated by the wide, large base of the blue triangle, and this also theoretically increases negative feedback (as indicated by the downward orange arrow. Four potential mechanisms are considered, as indicated in the blue boxes. These are predicted to increase negative feedback, or may provide mixed signals, as indicated for mechanisms 1–3, and for mechanism 4, respectively.

Potential mechanisms of action describing comparative differences between high and low (−) feedback cultures are shown in Figure 11.

Glutamate uptake (mechanism 1), is expected to remove glutamate from the extracellular space via amino acid transporters found on astrocytes (Danbolt, 2001). Secondly, removal of glutamate is expected to decrease [Ca^2+^]_i_ signaling, since glutamate increases calcium influx into cells (Figure 3). Third, the measured increased cell area for astrocytes (as indicated by GFAP staining) and the increased cell number in high (−) feedback cultures provide more surface area and volume for glutamate dissipation as a signal for any given cell. These first three potential mechanisms are all predicted to increase negative (−) feedback in measured [Ca^2+^]_i_ signaling. A fourth potential mechanism, that of modulation by glial cofactors, like ATP, is considered a mixed signal since ATP and glutamate may affect each other in complex ways. For example, it has been shown that ATP can be released from astrocytes triggered by elevation of intracellular Ca^2+^ (Lalo et al., 2014), and as we alter the ratio of astrocytes/neurons, this could alter cellular responses to glutamate and other stimuli in complex ways. As shown here, astrocytes, and endothelial cells of the brain (BMVECs), both respond to ATP addition itself by increasing [Ca^2+^]_i_ (Figures 8, 10).

## Conclusion

Astrocyte cells are key structural cells in the CNS which provide negative feedback through maintaining a balance in neuronal excitability. Understanding the importance of order of stimulations on mixed cultures can be an important step toward understanding the networks. We demonstrated that the order of stimulations using low subthreshold nanomolar concentrations of Glu is important, and the calcium response is not the same for different orders in the stimulus pairings. We have shown that the order of stimulation matters as far as how it is processed in these cell culture networks consisting of high or low levels of negative feedback, as controlled here using the anti-mitotic FdU (Hui et al., 2016). Pure astrocyte cultures and brain microvascular endothelial cells (BMVECs) demonstrated calcium influx in response to ATP stimulation, and in the case of co-cultures of BMVECs and astrocytes, increased astrocyte content provided (−) feedback to this response. In summary, networks with high negative feedback (many astrocytes) are simpler, presenting only 4 of the 8 possible response scenarios compared to low negative feedback (fewer astrocytes) which demonstrated responses in 6 of the 8 possible response types for repeated Glu stimulus (Figure 3). Additionally, pure astrocyte cultures stimulated with combinations of ATP or high Glu concentrations demonstrated immediate response to ATP addition as far as calcium responses but not to initial additions of Glu (Figure 8). However, initial Glu additions to these same glial cultures did then cause a huge number of increasing calcium oscillations in the glial networks which were then treated with ATP (Figure 8C). A minority of microglial cells (5%), was observed in pure astrocyte cultures (Wang et al., 2005), including in [Ca^2+^]_i_ imaging experiments after ionomycin stimulation (Supplementary Figure 3). Thus, for both primary cultures of brain cells with high and low negative feedback, the type of response as measured by calcium dynamics is shaped by the order of the particular stimulus. This is appealing in the sense that the CNS and the brain in general is made to sense changes and the order of the input thus matters in how those changes are therefore processed. Using this new information, we can expand this approach to other cell types and study the effects of other excitatory and inhibitory neurotransmitters such as dopamine and GABA on the order of cellular responses. We also confirmed that there is a possibility to determine the cell types in these mixed cultures due to size of the cells and stimulus choices. More studies may need to be performed to determine the exact cell type based on their response to excitatory stimuli.

We found that BMVECs produce excessive NO during inflammation that could potentially initiate epileptogenesis and neuronal cell death by forming toxic peroxynitrite. Such produced NO was significantly inhibited by the presence of astrocytes in the co-culture model thus suggesting the negative feedback effect of astrocytes to the positive feedback mechanism of NO synthesis in BMVECs during inflammation combined with the clear negative feedback data represented here for calcium imaging in neurons and BMVEC cells. For example, the inflammatory agent TNF was earlier found to produce excessive [Ca^2+^]_i_ influx in neurons and was indicated as a sign of calcium dysregulation caused by overexpression of voltage-sensitive calcium channels (Jara et al., 2007; Park et al., 2008; Sama and Norris, 2013). This [Ca^2+^]_i_ dysregulation may be a deciding factor for neuronal cell death and neuronal excitotoxicity, which could also be contributors toward epilepsy (Sama and Norris, 2013; Patel et al., 2017; Rana and Musto, 2018). In this study, we found astrocytes provided (−) feedback as reflected by measured [Ca^2+^]_i_ dynamics in both BMVEC- and neuronal- cultures.

Combined, the results shown here point to the central role of astrocytes in providing (−) feedback to an excitable brain. Excitation, while central to sensing change, can also lead to seizures, and mediators such as NO which increase blood flow, can also exacerbate epileptogenic conditions (Kovács et al., 2009; Sharma et al., 2019). As we have shown here, the presence of astrocytes can balance both the downstream signaling cascades of [Ca^2+^]_i_ increases and NO production, at least as expressed in these *in vitro* primary brain cell systems.

## Data Availability Statement

The raw data supporting the conclusions of this article will be made available by the authors, without undue reservation.

## Ethics Statement

The animal study was reviewed and approved by Louisiana Tech University Animal Care and Use Committee.

## Author Contributions

EK and MD designed, conducted, and analyzed the experiments related to Calcium Imaging. NP and MD designed, conducted, and analyzed experiments related to NO analysis. EK, NP, and MD wrote the manuscript. All authors contributed to the article and approved the submitted version.

## Conflict of Interest

The authors declare that the research was conducted in the absence of any commercial or financial relationships that could be construed as a potential conflict of interest.
