# Supplementary material for: Negative Feedback Role of Astrocytes in Shaping Excitation in Brain Cell Co-cultures
Source: Front Cell Neurosci. 2021 Jul 13;15:651509. doi: 10.3389/fncel.2021.651509 (PMC8313828; doi:10.3389/fncel.2021.651509)
Supplement: Supplementary file 1 [file Data_Sheet_1.PDF]

Supplemental File- Data Sheet-

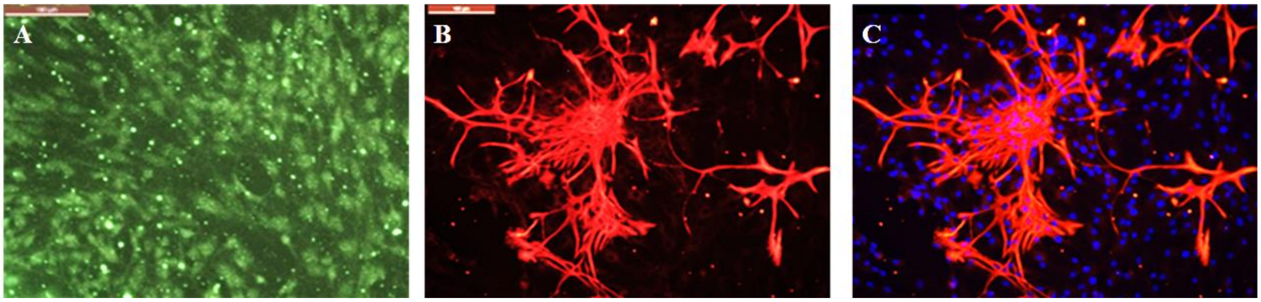

FIGURE S1 | **(A)** BMVECs stained against VWF **(B)** Astrocyte stained against GFAP **(C)** Co-culture Model-Astrocyte stained against GFAP (Red) amongst BMVECs' nuclei stained with DAPI (Blue), Magnification 20X, scale bar=100µm.

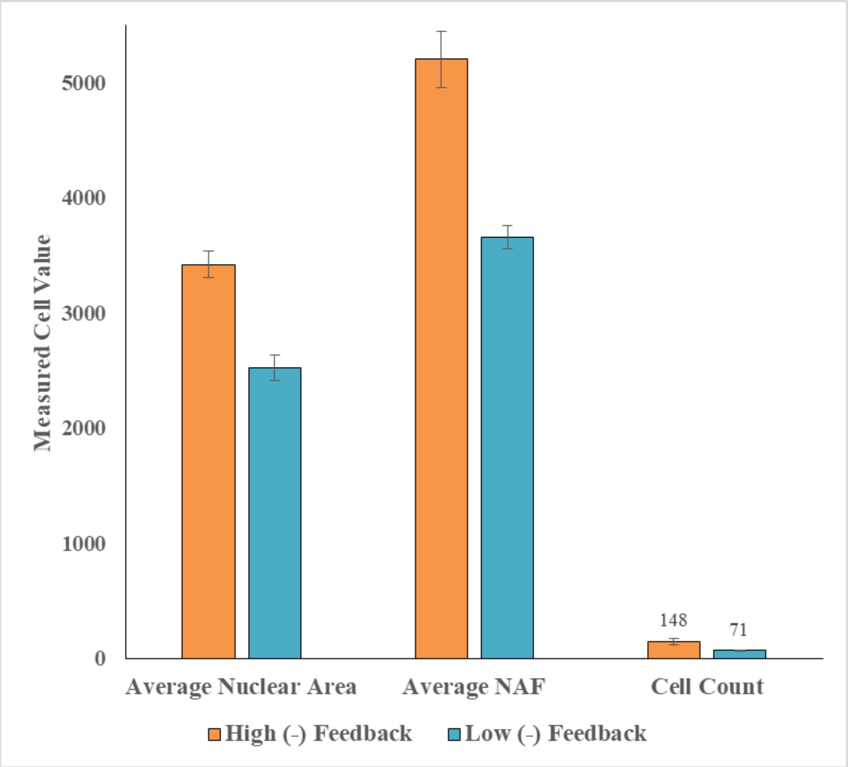

FIGURE S2 | Examples of comparative cell measurements in high and low (-) feedback cultures, as indicated. Three comparative measurements were made: Average nuclear area (measured in pixels^2), Average NAF (nuclear area factor, measured in pixels^2), and cell count are shown. Data are the average of 14 different fields from multiple wells of high and low (-) feedback cultures as indicated, with standard deviation error bars shown.

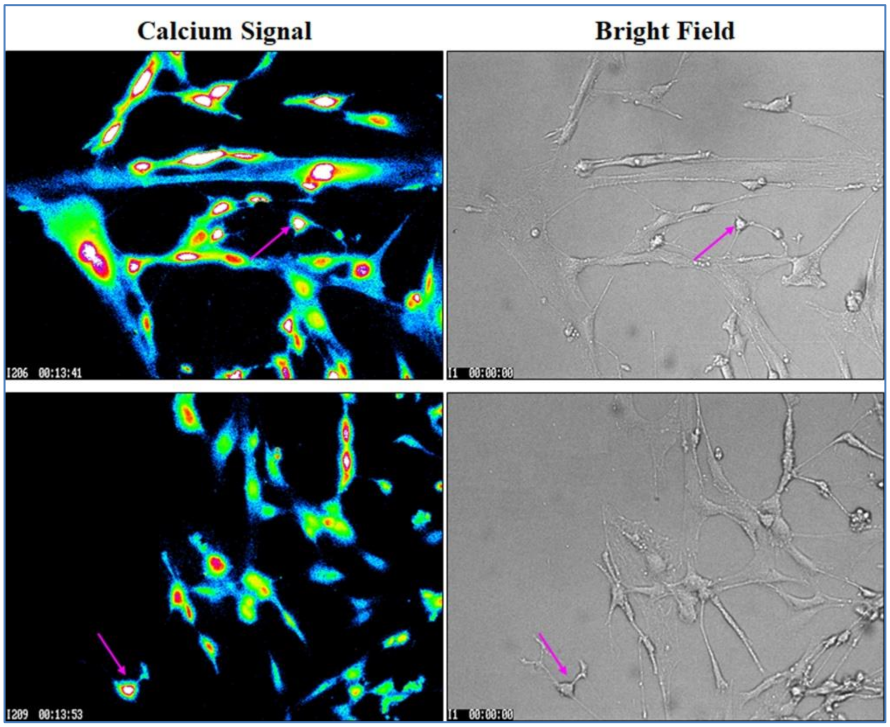

FIGURE S3 | Two examples of pure astrocyte cultures used for $[Ca^{2+}]_i$ imaging with minority population of microglial cells shown. Figures in the left column show pseudo-color calcium signal after maximal fluorescence stimulation with ionomycin. Figures in the right column show the bright field, monochrome transmitted light signal using the same calcium imaging system for the same cellular fields imaged for calcium. An example microglial cell is shown in each panel as indicated by the purple arrow.

Table 4 | Percentage of cells responding to different Events.

	**Only Event 2**		**Only Event 3**
**Low (-) feedback (Glu First)**	12.83	**Low (-) feedback (KCl First)**	0.77
**High (-) feedback (Glu First)**	28.97	**High (-) feedback (KCl First)**	1.25

Glu-First Events= Glu 250 nM, KCl 50 mM, Glu 500 nM, and Iono. KCl-First Events= KCl 50 mM, Glu 250 nM, KCl 50 mM, and Iono. Glu-First Low (-) feedback: n=577 cells; Glu-First High (-) feedback: n=1008 cells; KCl-First Low (-) feedback: n=781 cells; KCl-First High (-) feedback: n= 880 cells.
